# Anticancer Properties of Curcumin Against Colorectal Cancer: A Review

**DOI:** 10.3389/fonc.2022.881641

**Published:** 2022-04-22

**Authors:** Oluwafemi Adeleke Ojo, Temiloluwa Rhoda Adeyemo, Damilare Rotimi, Gaber El-Saber Batiha, Gomaa Mostafa-Hedeab, Matthew Eboseremen Iyobhebhe, Tobiloba Christiana Elebiyo, Bukola Atunwa, Adebola Busola Ojo, Clara Mariana Goncalves Lima, Carlos Adam Conte-Junior

**Affiliations:** ^1^ Phytomedicine, Molecular Toxicology, and Computational Biochemistry Research Laboratories, Department of Biochemistry, Landmark University, Omu-Aran, Nigeria; ^2^ Phytomedicine, Molecular Toxicology, and Computational Biochemistry Research Laboratories, Department of Biochemistry, Bowen University, Iwo, Nigeria; ^3^ Department of Pharmacology and Therapeutics, Faculty of Veterinary Medicine, Damanhour University, Damanhour, Egypt; ^4^ Pharmacology Department and Health Research Unit, Medical College, Jouf University, Sakaka, Saudi Arabia; ^5^ Pharmacology Department, Faculty of Medicine, Beni-Suef University, Beni Suef, Egypt; ^6^ Department of Physical Sciences, Chemistry Unit, Landmark University, Omu-Aran, Nigeria; ^7^ Department of Biochemistry, Ekiti State University, Ado-Ekiti, Nigeria; ^8^ Department of Food Science , Federal University of Lavras, Minas Gerais, Brazil; ^9^ Center for Food Analysis (NAL), Technological Development Support Laboratory (LADETED), Federal University of Rio de Janeiro (UFRJ), Cidade Universitaria, Rio de Janeiro, Brazil

**Keywords:** curcumin, colorectal cancer, pharmacological activities, bioactive compounds, anticancer

## Abstract

Colorectal cancer (CRC) is one of the most common and reoccurring diseases, as well as the world’s second largest cause of mortality. Despite existing preventative, diagnostic, and treatment methods, such as chemotherapy, the number of instances rises year after year. As a result, new effective medications targeting specific checkpoints should be developed to combat CRC. Natural compounds, such as curcumin, have shown significant anti-colorectal cancer characteristics among medications that can be used to treat CRC. These chemicals are phenolic compounds that belong to the curcuminoids category. Curcumin exerts its anti-proliferative properties against CRC cell lines *in vitro* and *in vivo via* a variety of mechanisms, including the suppression of intrinsic and extrinsic apoptotic signaling pathways, the stoppage of the cell cycle, and the activation of autophagy. Curcumin also has anti-angiogenesis properties. Thus, this review is aimed at emphasizing the biological effect and mode of action of curcumin on CRC. Furthermore, the critical role of these substances in CRC chemoprevention was emphasized.

## Introduction

CRC is one of the top causes of death worldwide, with an estimated 16.5 million deaths in 2015. It is one of the three most common diseases in males and ranked as the second top cancer in women after breast cancer (1). Colorectal cancer is also considered as an ailment for old people but it also causes threat for people under the age of fifty in recent times (2 *).* Colorectal cancer has been said to be commonly present in Western and Northern Europe and USA but uncommon in Africa, Asia, and India. Colorectal cancer is expected to increase by 60% in 2030 leading to a mortality rate of more than 1.1 million (1). The later stage diagnosis of CRC happens when metastasis occurs that is when the cancer has reached stage four. A lot of treatment modalities has been introduced but the side effect during and after treatment should be also considered (3, 4). Hence safer medications are to be enforced as a treatment method. Diets that have remedial possessions such as legumes, diet high in glycemic and curcumin (5).

Curcumin is known as a golden herb. It is an oily molecule soluble in acetic acid as well as ketone but appears to be insoluble in water (1). It contains features that cures cancer and it is also said to be a candidate for the cure of CRC. Curcumin has properties attributed to both anti-oxidant and anti-inflammation which contribute to its usage as a chemosensitizer (6). Curcumin a naturally occurring derivative of turmeric as well as a known bioactive compound. It is also a well-studied secondary metabolite with anti-cancer property (1). Curcumin nanoparticles have potent anti-cancer activity compared to free curcumin, which does not destroy normal cells (7). Curcumin targets a variety of apoptotic mechanisms including transcription factors among others. Curcumin alters the colorectal cancer stem cell growth *via* growth factor modulation, cell death, and epigenetic alteration (1). Hence, the major reason for this study is to emphasize the significance of curcumin as a source of anticancer medications against colorectal cancer by focusing on its pharmacological activities in chemoprevention.

## Colorectal Cancer Prevalence

It has been documented in developing countries that men have a higher risk of contracting this malignancy. It varies greatly by location, with up to an eightfold difference between countries; in emerging countries, incidence rate tends to increase in respect to increasing Human Development Index (HDI) (2). Colorectal cancer is the world second deadliest cancer accounting for 881,000 deaths in 2018. It is the deadliest among males in Saudi Arabian, Oman, and UAE and the deadliest among women in Algeria, Belarus, Spain, Portugal, Japan (2). The relationship between trends in prevalence and death can be divided into three main categories: semi-periphery nations, which has experienced an elevation in the occurrence and death in recent times and as a result of the economic transition they are undergoing; high HDI countries, which have seen an upsurge in occurrence but a decrease in death rate because of their management routes; and highest HDI countries, which have seen a reduction in both the occurrence and death rate because they have succeeded in preventing and treating colorectal cancer.

## Risk Factors of Colorectal Cancer

Risk factors of CRC can be split into two categories: modifiable risk and non-modifiable risk. We have the following modifiable risks: smoking, westernized food, physical inactivity, chronic diseases, and pharmaceuticals.

Smoking is responsible for 8.4% of colorectal cancer diagnoses and deaths in males. It has been established that it predisposes to rectal cancer and causes tumors with molecular abnormalities (8). Westernized diets which are known to contain less fruits, vegetables, and a larger portion containing processed meat and red meat which contribute to the growth of CRC (8). Colorectal cancer has been associated to a number of chronic conditions, comprising diabetes, hypertension, and coronary artery disease. Aside from controlling the body mass index (BMI) and other common variables, the risk of developing colorectal cancer is linked to the development of type 2 diabetes.

Race and ethnicity, sex, inherited mutations, gender, and body height are the non-modifiable risk variables. Differences in race and ethnicity are a major risk factor for CRC. It focuses on the disparities in accessing good health care, balanced diets, and education rather than the hereditary component. Males are 1.5 times as likely than females to acquire colorectal cancer (2).

## Managements of Colorectal Cancer

In recent years, surgery-based comprehensive treatment has become the mainstay of colorectal cancer treatment. Patients who receive neoadjuvant radiotherapy and chemotherapy prior to surgery may have their tumor stage reduced, their local recurrence and distant metastasis reduced, and their quality of life improved (2). Early colorectal cancer can be treated with endoscopic tumor resection, such as transanal endoscopic microsurgery, which reduces surgical complications and preserves organ function in the patient. In advanced situations, stent insertion, palliative excision of the tumor, or simultaneous removal of the combined liver and lung metastases can ameliorate patients’ clinical problems. These individuals may be able to live for a long time if they receive postoperative radiation and chemotherapy (8). Furthermore, tumor immunotherapy has advanced significantly and is now the fourth most popular therapeutic option for colorectal cancer. Irrespective of the current landmark recorded in diagnosing CRC and its treatment, the prognosis for CRC patients continues to be poor. Furthermore, the prevalence of side effects and toxicities during colorectal cancer procedures substantially restricts their clinical use (9).

## Curcumin

**Figure 1:**
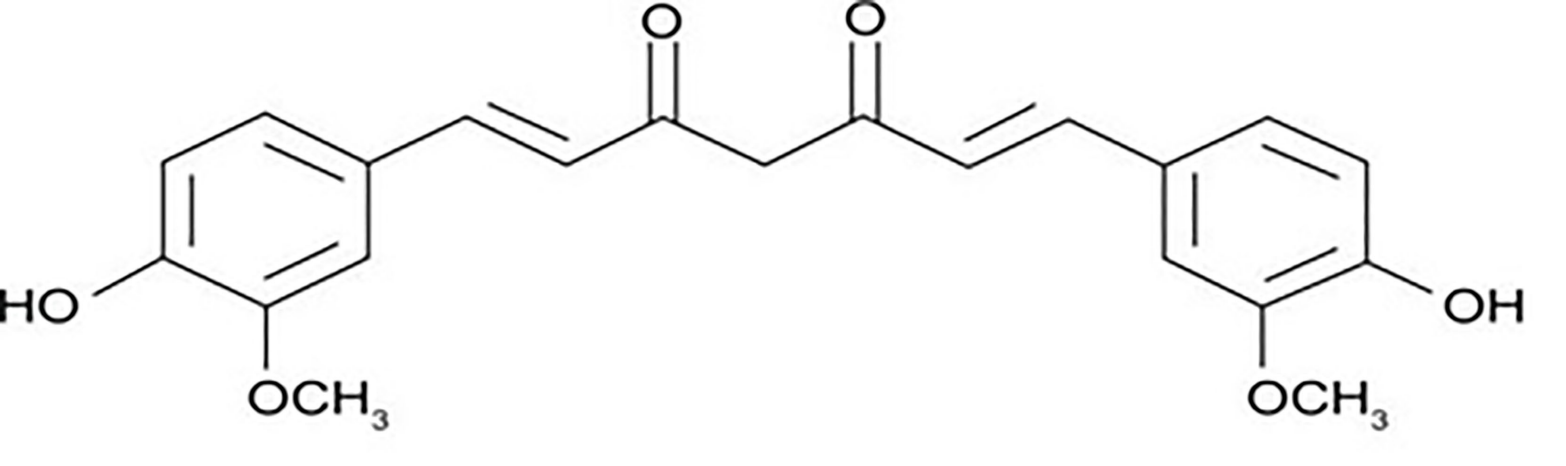
Structure of curcumin.

Curcumin was found as the most unique polyphenolic rhizome isolated from turmeric. Vogel and Pelletier isolated curcumin for the first time in 1815 while working in the Harvard College laboratory. It has been demonstrated to target several signaling molecules and to have biological function. Curcumin is utilized in a variety of forms for health benefits all over the world (10). **Figure 1** shows the structure of curcumin.

## Anti-cancer Activity of Curcumin

Curcumin has an important function in illness prevention by modulating biological processes.

It has an active role in pathogenesis prevention due to its efficiency as a radical scavenger. Curcumin is an efficient radical scavenger and its antioxidant activity has previously been proven by blocking the controlled beginning of styrene oxidation (11). Curcumin’s antioxidant activity, which controls DNA damage as well as lipid peroxidation caused by free radicals, is linked to its anticancer properties.

## Research Methodology

Data for this review on anti-colorectal cancer properties of curcumin were gathered from internationally recognized databases (scientific) through an electronic search (Wiley, SciFinder, Google Scholar, Springer, Web of Science, Francis & Taylor, Elsevier, PubMed, and The PlantList database). Furthermore, the medical books, PhD and MSc theses associated with the anti-colorectal cancer abilities of curcumin have been thoroughly researched.

## Anti-oxidant Activity

Curcumin improves the body’s oxidative stress markers. Antioxidant activity, such as superoxide dismutase, is thought to increase in serum. A meta-analysis of data selected at random, on the efficacy of the curcuminoids supplementation in oxidative stress revealed that the supplementation produced a considerable effect on every marker of oxidative stress analyzed, comprising superoxide dismutase and catalase activity in the plasma, as well as reduced glutathione (GSH) and lipid peroxides concentrations in the serum. Its influence on the radicals is controlled in different ways (12). Curcumin scavenges a wide range of free radicals, including ROS and also nitrogen species. It inhibits enzymes responsible for producing ROS which includes lipoxygenase/cyclooxygenase and xanthine hydrogenase/oxidase *via* altering the levels of GSH, catalase, and superoxide dismutase thereby neutralizing the created free radical (13). Curcumin, lipophilic molecule (Curcumin) like vitamin E, works as an effective scavenger of peroxyl radicals, making it a chain-breaking antioxidant (14).

## Anti-Inflammatory Activity

Many chronic diseases related to the pathogenic processes of oxidative stress, resembles those of inflammation such that one can easily induce another. In reality, inflammatory cells are known to generate a variety of ROS at the location where inflammation take place, resulting in oxidative stress, establishing a connection between oxidative stress and inflammation (14). Furthermore, a variety of ROS/nitrogen species can activate a cascade that signals intracellularly which promotes the activation of the genes responsible for pro-inflammation (14). Curcumin, the major component of turmeric, reduced inflammation and metabolism of arachidonic acid in the skin epidermis in an animal model study by downregulating the cyclooxygenase and lipoxygenase pathways, while another study discovered that curcumin has anti-inflammatory characteristics (11). Inflammation has been related to the emergence of a vast spectrum of chronic diseases for examples cardiovascular disease, cancer, metabolic syndrome Alzheimer’s disease (AD), diabetes, multiple sclerosis (MS), asthma, colitis, arthritis, psoriasis, diabetes, and Parkinson’s disease (PD) (15). Curcumin has also been found to reduce inflammation in ways that are not captured by this study, implying that it could be applied as an anti-inflammatory medicine (15).

## Anti-Tumor Activity

Many natural compounds originating from plant seeds, flowers, leaves, and stems have been researched in different studies and proven to be very helpful in preventing tumor development. Drug-metabolizing enzymes like cytochrome p450 and its reductase have been inhibited by the action of curcumin (11). Several animal studies discovered that curcumin, increase Phase II enzymes such as GSTs while decreasing the activity of VEGF (vascular endothelial growth factor) by inhibiting the receptor PPAR (peroxisome proliferator-activated) present in the colon cancer cells (11). Another study discovered a substantial decrease in cells viability because the cells were treated with curcumin, which correlates with the apoptotic activation linked to Notch-1 and NF-κB down-regulation (16). Curcumin, according to one study, can cause cell death and restrict the development of melanoma cells (17).

## Anti-microbial Activity

Globally, drug resistance to microorganisms is developing, and antimicrobial resistance is one of the leading causes of treatment failure. To address such issues, a natural source that is both safe and effective is necessary. Curcumin, has been proven to possess antibacterial, antiviral, and antifungal properties (18, 19). According to the findings, curcumin inhibited methicillin-resistant Staphylococcus aureus strains with a minimum inhibitory concentration of 125–250 g/Ml (20). Curcumin, one of turmeric’s key components, suppressed the growth of all Helicobacter pylori strains taken from patients that are infected with gastrointestinal symptoms *in-vitro* (21). Curcumin is an antibacterial molecule that kills Gram-positive and Gram-negative bacteria. Curcumin and its novel analogs, comprising; gallium-curcumin and Cu-curcumin, exhibit excellent anti-viral efficacy against HSV-1 in cell culture, according to the research (22).

## Immunomodulatory Properties

Immunomodulatory processes play an important function in the modulation of the immune system, either by enhancing or inhibiting immunological responses. Curcumin act as a major player in the immune system modulation (11). Curcumin was evaluated on T, B, and macrophages and found to suppress the immune system by down-regulating CD28 and CD80 expression while increasing cytotoxic T-lymphocyte antigen 4 (CTLA-4) expression (11). Curcumin reduced the proliferation of lymphocytes generated from fresh human spleen when they were exposed to phytohemagglutinin (PHA), phorbol-12-myristate-13-acetate, and concanavalin A. Curcumin suppresses NO generation, IL-2 production, and lipopolysaccharide-induced NF-κB while enhancing NK cell cytotoxicity (11). Curcumin has been demonstrated to play a crucial role in modulation of B-cells, macrophages, dendritic cells, T-cells, cell-cycle protein, humoral mediated immunity and cell-mediated in a number of prior studies (11).

## Anti-Proliferative Activity

Curcumin has been identified to have chemo-preventive and anticancer abilities when used alone or in combination, and has been used to treat and manage cancers such as colorectal, pancreatic, breast, prostate, lung, and oral (23–30). Curcumin suppresses the activity of NF-κB, activated protein-1 (AP-1), and epidermal growth response-1 (Egr-1), which are all implicated in cancer initiation and development. Most malignancies have certain factors that regulate transcription in enhancing cell proliferation, angiogenesis, and tumor development (31). Down-regulation of Bcl-2, Bcl-xL, COX-2, and matrix metalloproteinase (MMP)-9 are all affected by the NF-κB pathway, leading to cell inhibition, cell growth suppression, and cell death (32, 33). Curcumin has an impact on cancer cell proliferation *via* regulating AP-1 and STAT3. Cancer cells grow more slowly when both AP-1 and STAT3 are downregulated. Because it is prevalent in a wide range of solid tumors, including colorectal cancer, epidermal growth factor receptor (EGFR) is a major cancer therapeutic target. The EGFR is a significant target in cancer treatment. The reduction of growth in cancer cell invasion, and metastasis has been associated to EGFR downregulation (33). Curcumin has been demonstrated to suppress colon cancer cell development by lowering EGFR expression, which is mediated by a decrease in Egr-1 activity in Caco2 and HT29 colon cancer cells (34). Curcumin can trigger apoptosis by boosting the expression of the p53 gene, resulting in cell death during the G2 phase. When p53 was activated, it resulted in the reduction of anti-apoptotic genes Bcl-2/Bcl-xL and the overexpression of pro-apoptotic genes Bax, leading to cell death in the colon cancer cells (35).

## Curcumin Bioavailability

Curcumin’s clinical development is hampered by its low aqueous solubility and low bioavailability, despite its significant promise in the treatment of cancer. Curcumin was administered orally at a dosage of 8 g/day in clinical studies and it was found to undergo rapid biotransformation leading to a low amount of free curcumin in plasma (2.5 ng/mL) (36). Curcumin’s stability, solubility, and, most importantly, bioavailability has all been improved in recent years. Chemical alterations or chemical synthesis of curcumin analogues have been used as an approach for obtaining derivatives of curcumin. Because the oxyphenyl and carbon chain moieties appeared to be the important locations of the target for anti-tumor activity, several research have concentrated on structurally altering the aforesaid target sites, with promising results (37). Despite the fact that curcumin analogues are an effective method to improve curcumin bioavailability, many studies have been focused on developing unique delivery strategies to improve curcumin pharmacokinetics. Curcumin encapsulated in protein nanoparticles had improved anticancer action, as evidenced by MCF-7 cell viability loss and improved oral bioavailability in animal rat model (38). LipocurcTM (liposomal curcumin for infusion) and Meriva^®^, two potential nanocurcumin formulations, have been found to increase curcumin bioavailability and improve treatment results in patients with pancreatic and lymphocytic leukemia, respectively (39). Relevant results were found in Sprague–Dawley rats after oral therapy with exosomal curcumin (ExoCUR). ExoCur increased curcumin absorption and antiproliferative effectiveness when compared to free curcumin in an array of cancer cell lines (40). Antony et al. (41) evaluated the patented composition BCM-95^®^ CG (a mix of reconstituting curcumin with non-curcuminoid components of turmeric) on a group of human volunteers in order to measure curcumin bioavailability in blood. When compared to free curcumin, BCM-95^®^ CG (BiocurcumaxTM) increased relative bioavailability by 6.93 times and by 6.3 times when compared to a curcumin–lecithin–piperine mixture (41). Despite its low bioavailability, which is attributable in part to its chemical instability, many *in vivo* investigations, predominantly preclinical studies, continue to emphasize curcumin’s therapeutic properties, despite the fact that larger-scale placebo-measured trials are required to fully assess its impacts in humans.

## Anti-cancer Activity of Curcumin Against Colorectal Cancer

Curcumin is known to interrupt the cell-cycle as well as accelerate cell death, which can assist to inhibit the spread of colorectal cancer. According to *in vitro* investigations on multiple cancer cell lines, curcumin caused inhibited cell growth by reacting with several targets molecularly, leading to the control of multiple series of distinct signaling cascades. Curcumin inhibited cell growth by halting the cell-cycle present in the phase G2/M and partially in the G1 phase of the cancer cell line HCT-116, in the human colon cancer, according to Mosieniak et al. (42). Curcumin also inhibited cyclin D1 and caused cell-cycle disruption in the G1 phase of the same cancer cell line, according to (43). Cyclin D1 binds both CDK4 and CDK6, producing a very active complex that phosphorylates Rb protein at Ser780 and, controlling the transition from the G1 phase to the S phase (44).

MicroRNAs (miRNAs) have been the most extensively investigated and acknowledged as significant participants in CRC pathogenesis to far, owing to their ability to block the expression of several downstream genes, many of which play a vital role in carcinogenesis. The underlying mechanisms by which natural dietary substances, such as curcumin, alter the production of such short non-coding RNAs in CRC are of great interest. Curcumin therapy in CRC cells previously resulted in upregulation of miR-27a and miR-34a, which are well known for their tumor suppressive action in CRC (45). Curcumin has also been demonstrated to reduce invasion and metastasis in CRC patients by suppressing miR-21 expression (46), as well as miR-130a suppression, which prevents the Wnt/-Catenin signaling pathway from being activated. Curcumin treatment of CRC cells suppressed production of miR-27a, miR-20a, and miR-17-5p, leading in activation of ZBTB10 and ZBTB4, which are suppressors of specificity protein (Sp) transcription factors, according to a recent study (47). The upregulation of ZBTB10 and ZBTB4 expression inhibited SP transcription factors and, as a result, the expression of several downstream target genes such as EGFR, c-MET, cyclin D1, and NFB, resulting in cancer cell growth inhibition and apoptosis induction (47, 48). These findings emphasize curcumin’s epigenetic modulatory effects in CRC, providing mechanistic support for this botanical’s potential as a multi-targeted chemopreventive agent. (**Table 1**).

**Table 1:** Mechanism of action of curcumin in colorectal cancer therapy (45).

Experimental model (cell lines & animals)	Mechanism of action
HCT116, HCT116^p53-/-^, and SW480 cell lines (cell lines)	↓ cellular proliferation ↑induced apoptosis and cell-cycle arrest ↑miR-34a ↓miR-27a
HCT15 cells (cell lines)	↓proliferation ↑apoptosis ↓p53 and Prp4B
HCT116, HT29, HCT15, HCC2998, Colo205, Km12, and SW620 cells (cell lines)	↓migration, invasion, and colony formation *in vitro* cells ↓tumor growth and liver metastasis in mice model. ↓Sp-1, FAK ↑E-Cadherin
RKO and HCT116 cells (cell lines)	↓tumour growth, invasion and *in vivo* metastasis ↓miR-21
HCT116 cells (cell lines)	↑S and G2/M phase arrest ↑DNA damage
HCT116 and Caco-2 cells (cell lines)	↑G(2)/M stage arrest ↑mitotic spindle abnormalities and defects in chromosomal congression ↑DNA damage
RKO and SW480 cells (cell lines)	↑ROS, apoptosis ↓cell growth ↓SP1,SP3 and SP4
LoVo-xenograft (Animal model)	↑sensitivity to oxaliplatin, apoptosis ↑Bax, caspase-3, and PARP
HCT116-xenograft (Animal model)	↑radiosensitivity ↓NF-κB
Patient-derived colorectal liver metastases xenografts (Animal model)	↓cancer stem cell phenotypes ↑anti-proliferative and pro-apoptotic effects by FOLFOX treatment ↓number of ALDH^high/CD133-^cells
Orthotopically implanted CRC tumors (HCT116)- (Animal model)	↓growth and metastasis ↑sensitivity to capecitabine ↓NF-κB
DSS-induced tumor mice (Animal model)	↓disease activity index, ↓neoplasic lesions ↓ß-catenin, COX2, iNOS

## Curcumin Induces Apoptotic Signaling

**Figure 2:**
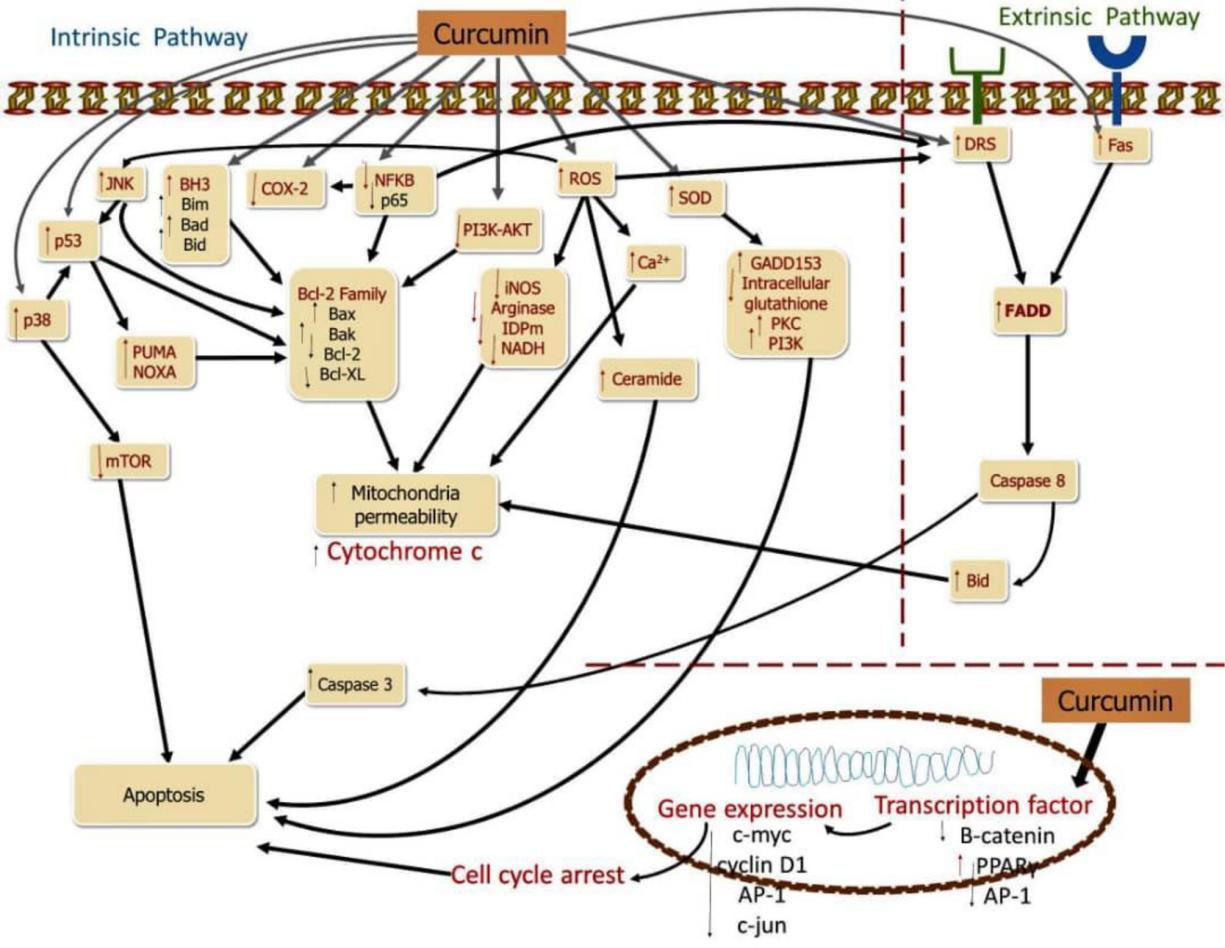
Curcumin induces apoptosis in CRC.

The activation of the cell death pathway is also another avenue by which curcumin perform its anti-cancer potential on CRC. Multiple molecular targets have been studied in the fundamental processes of curcumin incurring cell death in CRC (1). Changes in apoptosis-regulating cytokines are one of the pathways that lead to apoptosis disruption and resistance (27). The tumor necrosis factor ligand (TRAIL) family is a group of cytokines that function as apoptotic mediators. Binding of TNF-associated cell death initiating binding groups (Apo2L/TRAIL) and FasL23/CD95L ligands to precise membrane receptors responsible for pro-apoptotic of the TNF receptor family (receptor Fas/CD95, receptor DR4 and DR5) triggers the intracellular cell death *via* the extrinsic pathway. The death inducing signaling complex is started, when there is a binding between the ligand “death” and its reacting receptors, which then stimulates caspase 8, which triggers caspase 3 and therefore induces the death of cells (17). Caspase 8 may be activated by breaking Bid (a pro-apoptotic component of the Bcl-2 protein), leading to nonstop cytochrome C release. Extrinsic apoptosis regulation has been discovered to be related with immune system modulation in colon cancer cell lines, and it may be linked to the TRAIL and Fas signaling pathways (1). Overexpression of FasL, combined with downregulation of FasR expression and abnormalities in the Fas-mediated apoptosis signaling pathway, may result in the “death ligand” apoptotic pathway’s inactivation (28). CRC cells that have ligands that are not active may develop immunity, allowing them to evade cytotoxic immune system signals and penetrating the immune system, giving them a survival advantage and the ability to spread (1). The “Fas-counterattack hypothesis” could explain this occurrence (1). FasL expression was discovered at the early stages of the adenoma to carcinoma CRC process (1). Furthermore, most colon cancer cell lines with FasR positivity are resistant to Fas-mediated apoptosis, indicating a problem with the Fas-mediated signaling system (17) (**Figure 2**).

Curcumin causes to cell death in CRC cells, targeting several molecules and signaling pathways. Curcumin inhibits NF-κB cells as well as COX-2, decreasing catenin and stimulating activating protein-1 (AP-1), suppressing proteins responsible for anti-apoptotic processes while increasing ROS, SOD, and pro-apoptotic proteins, by up-regulating Fas. The main targets of apoptosis are shown by red molecules, while the downstreams targets of the red molecules are represented by black molecules (1).

## Mechanism of Action of Curcumin on Cancer Stem Cells

Colorectal cancer is a common type of cancer that affects people all over the world, and it is also the leading cause of death. Despite improved management options, the disease’s recurrence is the fundamental reason for its failure to be completely eradicated. Curcumin causes cell death by a variety of mechanisms, including targeting channels etc. Curcumin analogs are also being employed in clinical trials to improve bioavailability, solubility, and growth-inhibitor ability (49). To improve colorectal and brain cancer targeting, a range of technology based on nano materials, approaches have been established to overcome bioavailability and stability issues.

**Figure 3:**
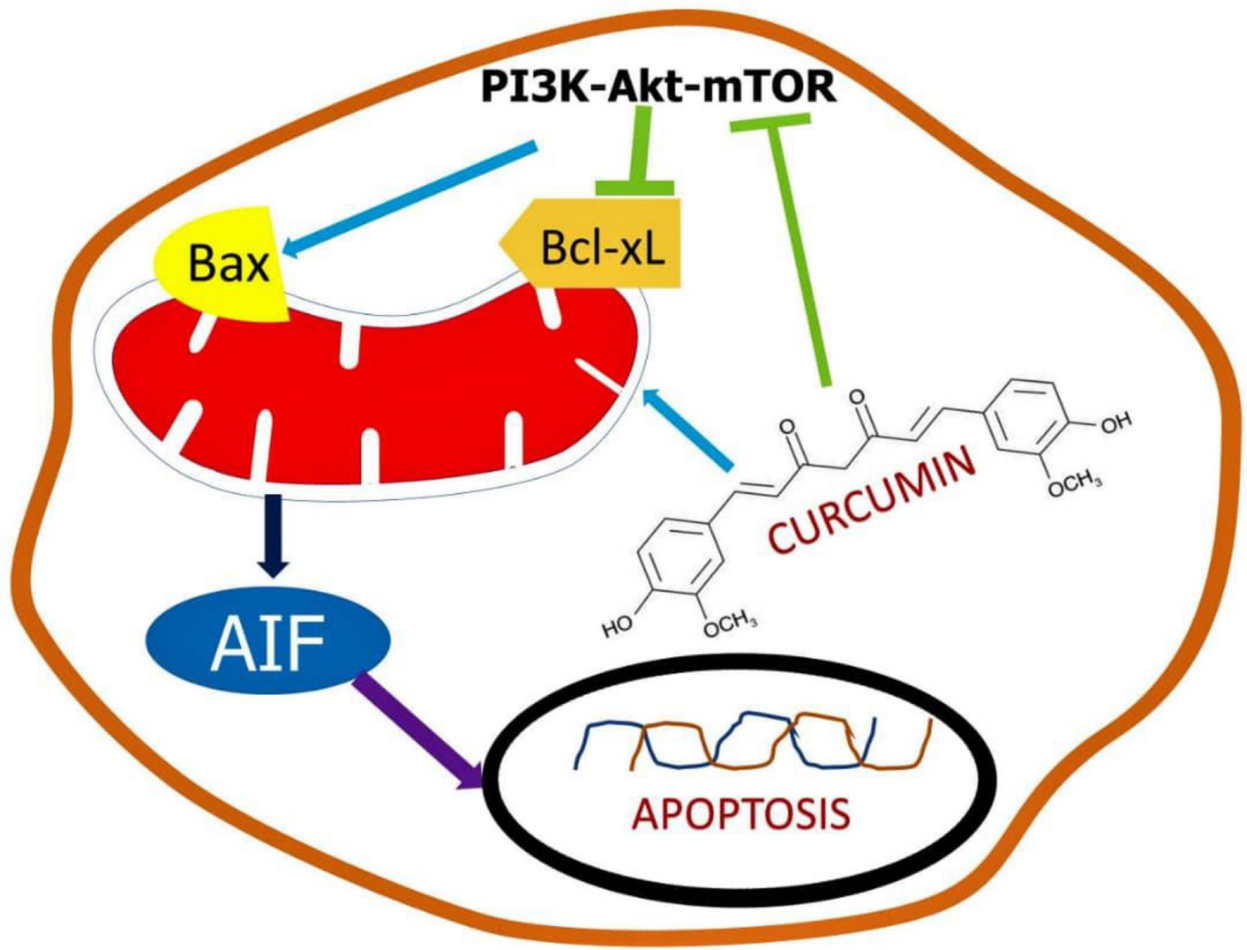
Schematic representation of the mechanism of curcumin in stem cells.

Evidence suggests that the existence of a minute number of cells known as cancerous stem cells causes medication resistance in colon cancer cells (CSC). Because some cancer stem cells have developed chemoresistance to regular treatment, overcoming tumor recurrence is a critical issue in colorectal cancer therapeutic management. Curcumin and its derivatives were demonstrated to be potent chemotherapeutic agents and chemosensitizers, with signs suggesting that their activity is mediated by microRNA regulation (50). Curcumin has been discovered to be a potent anti-proliferating agent both in the existence and nonappearance of synthetic drugs. The pathways responsible for the signaling is shown in **Figure 3**.

Curcumin can be an efficient chemo-therapeutic mediator and chemo-sensitizer on stem cells related to CRC by modulating a variety of pathways including apoptotic genes, signaling molecules, etc. This property was witnessed in *in vitro* cell line models as well as *in vivo* mouse models. Curcumin, through acting on the population of cancer stem cells (CSCs), can reduce tumor growth and chemoresistance cancer cells (51). The major limitation and failure in curcumin clinical research is owing to solubility difficulties, low absorption, and reduced the bioavailability of curcumin in tumor cells. New formulation options are being investigated and are in the early stages of development to tackle the challenges associated with poor bioavailability while also being effective in handling of CRC (52). Traditional therapy, on the other hand, does not address the resistance component; if chemo-resistance is not adequately handled, cancer cells will continue to grow and expand, eventually leading to metastasis. Patients’ survival odds will drop to 15–20 percent if cancer cells begin to spread. Traditional therapeutic approaches will then be ineffective in the treatment of cancer, even at high doses (53). The disadvantage of a large dose is that it causes several problems and undesirable effects. As a result, curcumin’s ability to inhibit the formation of CRC stem cells has been found beneficial in the treatment of cancer (54). Curcumin nanoparticles have been shown to be potent in overcoming pharmacokinetic and pharmacodynamic problems.

## Curcumin Nanoformulation in Colorectal Cancer

Curcumin distribution to the tumor location is improved by a nano formulation. Curcumin nanoparticles have been proven to exhibit significant anti-cancer activity when matched to free curcumin (7). Micelles, liposomes, nanogels, polymeric nanoparticles, are among the curcumin nano-formulations discovered by diverse researchers, according to mounting data.

## Curcumin Nanoparticles in Clinical Trials

The curcumin Nano formulation has been studied in three clinical trials for the management of colorectal adenomatous polyps and colorectal cancer (**Table 2**).

**Table 2:** Effects of curcumin on human clinical trials (55).

S/N	Study title	Condition	Intervention	No. of patients	Trial Phase	Current Status
1	First Line Avastin/FOLFIRI in Combination with Curcumin containing Supplement in Colorectal Cancer Patients with Unresectable Metastasis	CRC patients with unresectable metastasis	**Drug**: Avastin/FOLFIRI **Dietary Supplement**: Nanostructured lipid curcumin particles (100 mg)	50	2	Completed
2	A Prospective Evaluation of the Effect of Curcumin on Dose-limiting Toxicity and Pharmacokinetics of Irinotecan in Colorectal Cancer Patients	Advanced CRC patients	**Drug**: Irinotecan **Dietary Supplement**: Meriva^®^ (curcumin in a matrix of microcrystalline cellulose combined with soy lecithin Phosphatidylcholine)	23	1	Active
3	A Pilot, Feasibility Study of Curcumin in Combination With 5FU for Patients With 5FU-Resistant Metastatic Colon Cancer	5FU-Resistant Metastatic CRC patients	**Drug**: 5-flurorouracil **Drug**: BCM-95: micronized rhizome extract containing phospholipids and 500 mg of pure curcuminoids (95% curcumin, 5% desmethoxycurcumin) suspended in turmeric essential oil	13	1	Active
4	Phase I Clinical Trial Investigating the Ability of Plant Exosomes to Deliver Curcumin to Normal and Malignant Colon Tissue	CRC patients	**Dietary Supplement**: Curcumin **Dietary Supplement**: Curcumin conjugated with plant exosomes **Other**: No Intervention	7	1	Active
5	Phase I Pharmacokinetic Trial of Curcuminoids Administered in a Capsule Formulation	CRC patients	**Dietary Supplement**: Curcuminoids in capsule formation	N/A	1	Completed
6	Randomized Window of Opportunity Trial of Anthocyanin Extract and Phospholipid Curcumin in Colorectal Adenoma	Subjects With Patients with colorectal adenoma	**Dietary Supplement**: Mirtoselect^®^ + Meriva^®^ **Dietary Supplement**: Placebo	100	N/A	Active

N/A, not applicable.

1. Curcumin conjugated plant exosomes aid in the transport of curcumin colon tumors and tissues. Exosomes have the ability to bind to hydrophobic medicines to as well as curcumin. Three groupings of subjects have been established. The first group were given plant exosomes (0.0036 kg) combined with curcumin in the form of a pill once a day for a seven days’ period. The second group receives only curcumin (0.0036 kg). The third category receives no assistance. The first result was expressed as the concentration of both curcumin in normal and cancer cells. The second objective was safety, which included differences in metabolic features as well as the immunological response to curcumin-loaded exosomes and curcumin. (NCT01294072; NCT01294072; NCT01294072; NCT01294072; NCT01294072 Panaro and colleagues (56).

## Curcumin on Human Colorectal Cancer

The impact of curcumin has not gone unnoticed in the management of CRC in humans and in the light of these several clinical trials have been done, some still ongoing below is a **Table 2** showing some of the clinical trials that are completede, and still ongoing.

2. Curcumin phytosome. According to the findings of a Phase II clinical trial, Meriva is a proprietary curcumin formulation containing lecithin (food-grade). The interventions make use of anthocyanin, mirtoselect, and Meriva. The goal of the study is to see if protein expression is necessary for the incidence of colon cancer in the treated group versus the placebo group. For 28 days, patients with colorectal cancer were administered 1 g of Meriva and Mirtoselect two times in a day. A significant observation was the difference in expression of -catenin biomarker in cancer as well as normal rectum mucosa. As a secondary result, the immunological histochemical appearance of Ki-67, NF-K, and p53 was evaluated. (NCT01948661, NCT01948661, NCT01948661, NCT01948661, NCT01948661, NCT01948661, NCT01948661, NCT01948661, NCT01948661, NCT01948661, NCT01948661, NCT01948661 (57).

3. Nanostructured lipid curcumin particles were found to be effective as a food supplement in addition to standard chemotherapy treatment in a separate Phase III clinical investigation. Folic acid plus avastatin, fluorouracil, and irinotecan (FOLFIRI) is a treatment that is given for days. Curcumin is also administered as an oral supplement (100 mg/day dose) until the treatment is finished. Curcumin particles having a lipid nanostructure were thought to improve curcumin absorption. The participants were then followed for two years after the intervention to see if they had progressed free of disease. Secondary outcomes were the long-range of response and survival rate, as well as protection (54).

## Impact of Curcumin on Gut Microbiota

CRC that is well known as one of the top three major types of cancer present in males and classified as one of the top two in females considering close to 2 million new cases as far back as 2018 (58). Some factors have been put into consideration in the likelihood of an individual developing CRC such as environmental and genetic factors which includes old age, obesity, chronic inflammation over a long period, as well as sedentary lifestyle and eating unhealthy foods (45). Curcumin, also known as diferuloylmethane, has been used in time past as spices in food. It is present in the root of *Curcuma longa* (turmeric). Its chemical structure, which has double bonds, makes it an effective electron donor which uses that ability to destroy the formation of reactive oxygen species in lots of redox reactions, which in turn becomes a potent antioxidant agent (59)

We have identified curcumin as having the ability of interrupting the gut microbiome (a microbe capable of influencing the progression of CRC). The species of gut microbes play a vital role in the structural, physiology, as well as performing basic metabolic and protective function on the health of the host (45). Any disturbance observed in the gut microbiome dysbiosis, can have serious outcomes leading to a risk of developing a variety of diseases such as CRC. Studies in humans have shown the ability of curcumin to tilt the ratio of pathogenic microbes and beneficial microbes. Curcumin, in fact, may lower intestinal inflammation *via* regulating gut flora. Curcumin was showed to reduce NF-κB activation in colonic epithelial cells and increase the growth of CD4+ Foxp3+ regulatory T cells in the colonic mucosa in an experimental DSS-colitis model (60).

Another study revealed the effect of curcumin consumed in diet could cause an increase in the species of *Clostridium*, *Enterobacter* (have the potential to enhance mucosal Treg cells by producing butyrate) and reducing the levels of *Blautia* and *Ruminococcus s*pecies (which has been linked with individuals that has CRC) that is present in abundance (45).

## Curcumin’s Significance in Cancer Prevention

The *Curcuma longa* roots (dried) plant are used to produce curcumin. It has been discovered to have anti-carcinogenic properties (61). Curcumin has the ability to target colon cancer cells specifically, while allowing normal cells to remain alone; cancerous cells die as a result of an elevation in the expression of a protein known as GADD45a (Gene activated during DNA damage). Normal cells are unaffected because the protein activation association is not triggered. Curcumin and other natural and manmade compounds are currently being researched in attempt to increase their anti-oxidant, anti-cancer, and anti-inflammatory potentials (62).

## Challenges in the Clinical Utilization of Curcumin in CRC Treatment

Curcumin has been explored in clinical trials to treat advanced colorectal cancers, however, there are limitations to the clinical utilization of curcumin as a therapeutic agent. Curcumin has a very low therapeutic window, owing to its poor solubility, poor absorption and rapid metabolism and rapid elimination (63, 64). The poor absorption and rapid metabolism of curcumin severely limit its bioavailability and effective delivery to target sites (65).

According to Karthika et al. (4), when 1g of curcumin was orally administered Sprague-Dawley rats, 75% of the curcumin was excreted unchanged, suggesting that curcumin is poorly absorbed when administered orally. Reports from a clinical trial conducted by Sharma et al. (66), revealed that curcumin was not detected in the blood and plasma of all the CRC patients administered 20 mg of curcuminoids and 200 mg of Curcuma essential oils, following daily oral administration for 29 days; however, a considerable amount of curcumin and curcumin sulphate was detected in their feces.

To circumvent this challenge, curcumin-based nano formulations and structural analogues of curcumin have been designed and explored in the treatment of colorectal cancers in preclinical studies (67, 68). According to Sorasitthiyanukarn et al. (69), nanoencapsulation of curcumin in chitosan/alginate nanoparticles could facilitate the controlled release of curcumin and enhance its cellular uptake into the gastrointestinal tract. Sufi et al. (70), reported that the loading indole-incorporated curcumin analogue and curcumin onto Polysorbate 80-stabilized PLGA nanoparticles preserved curcumin from degradation in wide ranges of pH environments. The loading of soluble curcumin onto pectin and skimmed milk powder dual layered solid lipid nanoparticles improved that stability of curcumin and sustained its release in different gastro-intestinal environment for up to 72h (71) (**Table 3**).

**Table 3:** Curcumin-based nano formulations and structural analogues in the treatment of colorectal cancers in preclinical studies.

S/N	Therapeutic agent	Type of study	Findings	Reference
1	Whey protein encapsulated curcumin	*In-vitro*	encapsulation of curcumin with whey protein at different ratios increased the intracellular bioavailability of by 12 -21% in human colon and prostrate cancer cell lines	Jayaprakasha et al. (68)
2	Orally deliverable nanotherapeutic engineered from water soluble curcumin and 7-ethyl-10-hydrocamptothecin (SN38)	*In-vivo*	The combinatorial therapy demonstrated remarkable tumor shrinkage in the CAC mice by inducing cell cycle arrest	Han et al. (72)
3	Curcumin diethyl diglutarate-loaded chitosan/alginate nanoparticles	*In-vitro*	Enhanced cellular uptake and, *in-vitro* digestibility and inaccessibility under stimulated gastrointestinal conditions	Sorasitthiyanukarn et al. (69)
4	hydrazinocurcumin derivative in Chitosan (CS), ZnO, Au, CS-ZnO and CS-Au-NPs	*In-vitro*	higher activity against HCT-116 cell lines	Kandile et al. (73)
5	Polysorbate 80-stabilized PLGA- loaded curcumin and indole-incorporated curcumin analogue	*In-vitro*	preserved curcumin from degradation in wide ranges of pH environments.	Sufi et al. (70)
6	Pectin and skimmed milk powder dual layered solid lipid nanoparticles loaded with soluble curcumin	*In-vitro*	improved that stability of curcumin and sustained its release in different gastro-intestinal environment for up to 72h	Moideen et al. (74); Mohamed et al. (71)
7	Curcumin- loaded dendrimer gold hybrid structure	*In-vitro*	Higher cellular cytotoxicity in comparison with free curcumin	Wong et al. (54)

Different ratios of whey protein loaded with curcumin was reported to possesses a significantly higher cytotoxicity against human colon and prostate cancer cell lines (IC_50_ = 16.86 - 30.91 µM) as compared with free curcumin (IC_50_ = 50 µM) (68). According to Jayaprakasha et al. (68), the encapsulation of curcumin with whey protein at different ratios increased the intracellular bioavailability of by 12 -21%, this enhanced curcumin’s ability to regulate proapoptotic proteins such as: Bax, p53 and cytochrome C (**Table 3**).

Encapsulation of chemically synthesized hydrazinocurcumin derivative in chitosan (CS), ZnO, Au, CS-ZnO and CS-Au-NPs, revealed higher activity against HCT-116 cell lines (73). Han et al. (72), administered the chemically engineered an orally deliverable nanotherapeutics from water-insoluble curcumin (CUR) and 7-ethyl-10-hydrocamptothecin (SN38) to colitis-associated colorectal cancer (CAC) mice and they observed that the combinatorial therapy showed remarkable tumor shrinkage in the mice.

## Conclusion and Future Perspectives

Finally, several studies show that curcumin belongs to a class of plant-derived chemicals that can help prevent colorectal cancer. Several possible pathways have been proven in both *in vitro* and *in vivo* animal studies. Furthermore, treatment improvements in animals with inflammatory and hereditary colorectal cancer have been discovered. Curcumin was given orally, either singly or in combination with chemicals or little elements that aid in transport and absorption. Curcumin is also used as an ingredient in plant-based dietetic formulations. This personality feature has a lot of potential in people. Despite this, no convincing outcomes from cultured cells or experimental animal models and be generalized to humans. Human clinical trials, on the other hand, are scarce and have produced contradictory results. Future large-sample trials, on the other hand, will be necessary to resolve unanswered questions concerning dose, bioavailability, proper indication, and potential toxicity.

## Author Contributions

OO, AO, TA, and DR conceptualized and designed the study, edited the main text and approved the final edition of the manuscript. TA, TE, BA, and MI performed the search, prepared the tables, wrote the main text, and approved the final edition of the manuscript. AO summarized information, and prepared the tables. OO, MI, and AO helped to organize the literatures and revise the manuscript. CL, C-CJ, GE-SB, and GM-H did the technical editing, review and editing of the manuscript. CL, C-CJ, GE-SB, and GM-H acquire funding for the manuscript. All authors approved the final edition of the manuscript.

## Conflict of Interest

The authors declare that the research was conducted in the absence of any commercial or financial relationships that could be construed as a potential conflict of interest.

## Publisher’s Note

All claims expressed in this article are solely those of the authors and do not necessarily represent those of their affiliated organizations, or those of the publisher, the editors and the reviewers. Any product that may be evaluated in this article, or claim that may be made by its manufacturer, is not guaranteed or endorsed by the publisher.
